# Insulin Resistance Exacerbates Alzheimer Disease via Multiple Mechanisms

**DOI:** 10.3389/fnins.2021.687157

**Published:** 2021-07-19

**Authors:** Zenghui Wei, Jagadish Koya, Sandra E. Reznik

**Affiliations:** ^1^Department of Pharmaceutical Sciences, St. John’s University, New York, NY, United States; ^2^Department of Pathology, Albert Einstein College of Medicine, New York, NY, United States; ^3^Department of Obstetrics and Gynecology and Women’s Health, Albert Einstein College of Medicine, New York, NY, United States

**Keywords:** Alzheimer’s disease, insulin resistance, amyloid beta, tau, drug

## Abstract

Alzheimer disease (AD) is a chronic neurodegenerative disease that accounts for 60–70% of dementia and is the sixth leading cause of death in the United States. The pathogenesis of this debilitating disorder is still not completely understood. New insights into the pathogenesis of AD are needed in order to develop novel pharmacologic approaches. In recent years, numerous studies have shown that insulin resistance plays a significant role in the development of AD. Over 80% of patients with AD have type II diabetes (T2DM) or abnormal serum glucose, suggesting that the pathogenic mechanisms of insulin resistance and AD likely overlap. Insulin resistance increases neuroinflammation, which promotes both amyloid β-protein deposition and aberrant tau phosphorylation. By increasing production of reactive oxygen species, insulin resistance triggers amyloid β-protein accumulation. Oxidative stress associated with insulin resistance also dysregulates glycogen synthase kinase 3-β (GSK-3β), which leads to increased tau phosphorylation. Both insulin and amyloid β-protein are metabolized by insulin degrading enzyme (IDE). Defects in this enzyme are the basis for a strong association between T2DM and AD. This review highlights multiple pathogenic mechanisms induced by insulin resistance that are implicated in AD. Several pharmacologic approaches to AD associated with insulin resistance are presented.

## Introduction

Alzheimer disease (AD) is a chronic degenerative brain disease characterized by memory loss, cognitive impairment, and loss of activities of daily living (Jha et al., 2019). It is the most common form of dementia and the sixth leading cause of death in the United States (Wilson et al., 2012; Heron, 2013). An estimated 5.8 million Americans suffered from AD in 2020 and this number will triple to nearly 14 million people by 2060 (Matthews et al., 2019). There are no treatments that effectively stop or reverse AD progression, although some medications temporarily improve symptoms (Hsu and Marshall, 2017). Notably, the United States Food and Drug Administration (FDA) approved Aducanumab on June 7th, 2021, the first antibody for the treatment of AD which reduces amyloid plaques. However, this drug had previously failed to gain FDA approval, because initial analysis of clinical trial data did not show a significant improvement in patients’ mental abilities. Phase IV trials are still required to verify its clinical benefits.

There are two major forms of AD: the sporadic (late-onset) form, which accounts for most cases, and the familial (early-onset) form, which is generally associated with the inheritance of genetic mutations (Bekris et al., 2010). While the cause of most AD cases is poorly understood (Reitz and Mayeux, 2014), genes encoding amyloid precursor protein (APP), presenilin 1 and presenilin 2 account for the majority of early-onset familial AD cases (Cheignon et al., 2018), whereas apolipoprotein E (APOE) is the main genetic risk factor in sporadic AD, especially *APOE−ε4* (Morris et al., 2014; Clark and Vissel, 2018).

The pathogenesis of AD is multifactorial (Crous-Bou et al., 2017). Accumulating studies indicate a strong association between type II diabetes (T2DM) and AD (Kang et al., 2017). Neuronal insulin signaling pathways are disrupted in both T2DM and AD and over 80% of AD patients have T2DM or display abnormal blood glucose levels (Zhao and Townsend, 2009). Observational studies demonstrate that T2DM nearly doubles the risk of AD and increases the likelihood of dementia (Leibson et al., 1997; Luchsinger et al., 2001; Xu et al., 2009). In addition, APOE4 and insulin resistance were found to impair cognitive function in a study of human E4-targeted replacement mice (Johnson et al., 2017). Multiple studies have also established that insulin resistance leads to the progression of two main pathological hallmarks of AD—senile plaques from extracellular deposition of amyloid β-protein and tau-based neurofibrillary tangles (NFT) (Ardura-Fabregat et al., 2017). Consequently, AD may be considered a type of metabolic disease, and the development of AD therapeutics may benefit from an understanding of the relationship between AD and insulin resistance (Kang et al., 2017).

## Insulin Resistance and AD

Insulin is essential for metabolic homeostasis in the peripheral system (Tokarz et al., 2018), but has only been recognized for its role in regulating amyloid β-protein peptides and the generation of NFTs in the last few decades (Razay and Wilcock, 1994; Kroner, 2009). Under normal conditions, increased plasma glucose levels lead to stimulation of pancreatic β-cells to produce insulin, which decreases glucose levels. As blood glucose falls, counter-regulatory hormones including epinephrine, norepinephrine and cortisol from the adrenal glands arrest insulin-mediated glucose disposal. Insulin is then rapidly degraded in the liver, kidney and muscles by insulin degrading enzyme (IDE) (Watson and Craft, 2003). The pleiotropic biologic effects of insulin are mediated via binding and activating insulin receptors (IR) (Boucher et al., 2014), which are widely distributed in the periphery but selectively distributed in the central nervous system (CNS), including the cerebral cortex, hippocampus, hypothalamus and amygdala (Havrankova et al., 1978; Bosco et al., 2011; Soto et al., 2019). Insulin binding leads to a conformational change of the IR resulting in phosphorylation of intracellular IR substrate (IRS) proteins on tyrosine residues (Saini, 2010). Subsequently, IRS activates downstream pathways including mitogen-activated protein kinase (MAPK) and phosphatidylinositol-3-kinase (PI3K) (Gabbouj et al., 2019), which are important for mitogenic and metabolic functions (Plum et al., 2005).

However, in insulin resistance, cells fail to respond to insulin causing elevated blood glucose and effects on muscle, liver and brain (Kroner, 2009; Zhao and Townsend, 2009). Pancreatic β-cells produce more insulin in response to high blood glucose (hyperglycemia) resulting in hyperinsulinemia (high blood insulin), eventually leading to T2DM (Heydemann, 2016). Decreased levels of insulin and IR are found in the cerebrospinal fluid (CSF) of AD patients due to long-term peripheral hyperinsulinemia and decreased insulin transport across the blood-brain barrier (BBB) (Craft et al., 1998; Rivera et al., 2005; Steen et al., 2005; Gil-Bea et al., 2010; Stanley et al., 2016).

**FIGURE 1:**
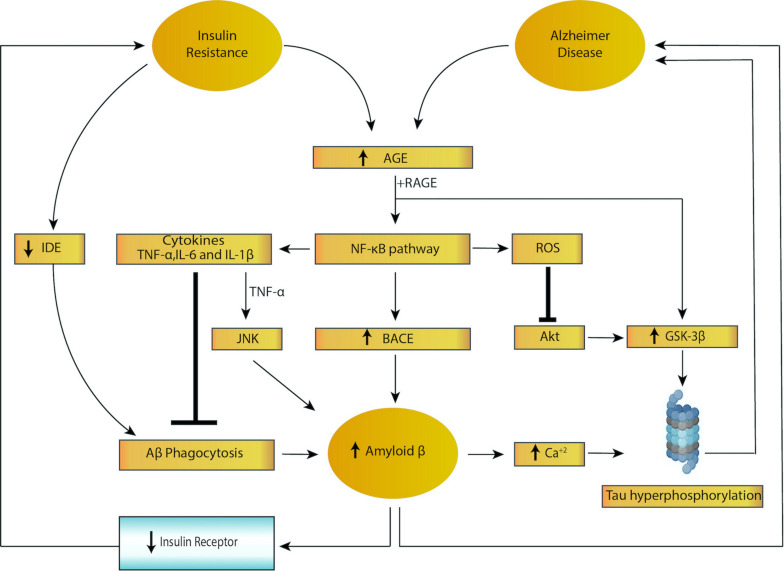
Feed forward loop of insulin resistance and Alzheimer disease. Both insulin resistance and Alzheimer disease lead to activation of nuclear factor kappa B (NF-κB), increased cytokine secretion and increased reactive oxygen species (ROS) levels, triggering increased amyloid beta (amyloid β) and tau hyperphosphorylation. In addition, insulin resistance lowers levels of insulin degrading enzyme (IDE), resulting in impaired amyloid β phagocytosis. Higher levels of amyloid β, in turn, leads to decreased expression of the insulin receptor, which results in insulin resistance, creating a vicious cycle.

Accruing evidence shows that insulin facilitates memory and cognition under normal conditions (Watson et al., 2009; Tokarz et al., 2018) whereas chronic hyperinsulinemia impairs them (Lee et al., 2016). For instance, fructose-induced insulin-resistant rat models show impaired spatial learning in the water-maze test (Sachdeva et al., 2019). Moreover, intranasal insulin improves memory in humans (Benedict et al., 2008; Krug et al., 2010). Insulin resistance may accelerate the progression of senile plaques and NFTs via multiple mechanisms, resulting in cognitive decline, impaired long-term potentiation (LTP) and associated metabolic disease. A summary of the feed forward loop of insulin resistance and AD pathogenesis is provided in Figure 1.

### Neuroinflammation Induced by Insulin Resistance in AD

The current consensus is that neuroinflammation plays a pivotal role in AD progression (Wang W. Y. et al., 2015), which is supported by results from APP transgenic mouse models in which injection of lipopolysaccharide (LPS, TLR4 activator) triggers neuroinflammation with two cellular hallmarks of AD in the brain, amyloid β-protein deposition (Lee et al., 2008; Go et al., 2016) and tau hyperphosphorylation (Kitazawa et al., 2005; Lee et al., 2010). Amyloid β-protein is the product of consecutive cleavage of APP by enzymes β-secretase (BACE) and γ-secretase. Processing of APP yields multiple forms of the protein; the 40 and 42 amino acid residue products are the most common forms (O’Brien and Wong, 2011). High levels of monomeric amyloid β-protein have a propensity to aggregate into fibrils and then plaques, resulting in neurodegeneration and induction of tau pathology (Mouchlis et al., 2020).

Inflammation is involved in activation of microglial cells, which are primarily responsible for amyloid β-protein phagocytosis. Microglia are brain-resident immune cells responsible for promoting phagocytotic clearance as well as providing trophic support to ensure tissue repair and cerebral homeostasis (Sarlus and Heneka, 2017). They also play a role in higher cognitive functions, such as learning and memory in the adult brain, and are involved in the pathogenesis of neurodegenerative diseases like AD. In the early stages of AD, activated microglia repair damaged tissue and decrease amyloid β-protein accumulation. However, chronic microglial activation induced by inflammation leads to release of inflammatory mediators and accumulation of danger-associated molecular patterns (DAMPs), which limits amyloid β-protein clearance, leading to more plaque accumulation, neuronal dysfunction and death (Clark and Vissel, 2015; Wang W. Y. et al., 2015; Brabazon et al., 2018). This hypothesis is supported by a longitudinal study showing increased levels of microglial activation in both mild cognitive impairment (MCI) and AD patients compared to controls, but a reduction in microglial activation following an initial peak in MCI patients (Fan et al., 2017). These data suggest that early microglial activation leads to a protective phenotype which can later turn into a pro-inflammatory picture due to failure of amyloid β-protein clearance and progressive neuronal damage.

Insulin resistance results in microglial activation and inflammation (McCaulley and Grush, 2017) by inducing the activation of resting (ramified) microglia and changes in cellular morphology, surface phenotype, secretary mediators and proliferative responses (Sarlus and Heneka, 2017). One common molecular pathology shared by insulin resistance and AD is increased levels of advanced glycation end products (AGEs) (Zhao and Townsend, 2009). Binding of AGEs to their cellular receptors (RAGE) not only upregulates glycogen synthase kinase 3β (GSK-3β), causing tau hyperphosphorylation (Peng et al., 2007; Li et al.,2012a,b), but also activates the NF-κB pathway, which produces reactive oxygen species (ROS) and pro-inflammatory cytokines [interleukin (IL)-6, IL-1β, TNF] (Kandimalla et al., 2017). These cytokines are observed to increase accumulation of amyloid β-protein in AD by two mechanisms: (1) increased levels of pro-inflammatory cytokines inhibit phagocytosis of amyloid β-protein in AD brains thereby hindering the removal of plaque by resident microglia; (2) TNF has been shown to upregulate the production of amyloid β-protein via activation of the c-Jun N-terminal kinase (JNK)-dependent MAPK pathway, which promotes phosphorylation and cleavage of APP (Liaoi et al., 2004; McAlpine and Tansey, 2008; Colombo et al., 2009; Montgomery et al., 2011; Cheng et al., 2014; Ahn et al., 2016; Decourt et al., 2017; Zhang et al., 2019). In addition, activation of the NF-κB pathway further increases BACE expression, resulting in increased production of amyloid β-protein (Guglielmotto et al., 2012; Cai et al., 2016). High levels of amyloid β-protein cause IR downregulation via internalization, desensitization or direct substrate competition, which ultimately turn into insulin resistance (Xie et al., 2002; Mullins et al., 2017). Moreover, amyloid β-protein triggers Ca^2+^ influx, which not only causes hyperphosphorylation of tau protein (Bosco et al., 2011) via GSK-3β, but also inhibits IR tyrosine kinase signaling. The increased levels of Ca^2+^ stimulate Ca^2+^-dependent serine/threonine protein kinases (PKC, Akt), which phosphorylate IRs and insulin resistance substrate (IRS) and thus negatively regulate IRs in the brain (Zhao and Townsend, 2009). Taken together, insulin resistance, neuroinflammation and exacerbation of amyloid β-protein and tau form a feed-forward loop in AD pathogenesis. Imbalance induced by any of these factors will facilitate AD progression, resulting in neurotoxicity, neurodegeneration and induction of a negative effect on IRs.

### Oxidative Stress Induced by Insulin Resistance in AD

Growing evidence suggests that insulin/insulin-like growth factor (IGF) signaling is strongly associated with oxidative stress. Brain insulin/IGF resistance may contribute to impairments in glucose utilization and disruption of energy metabolism, resulting in production of ROS, DNA damage and mitochondrial dysfunction, eventually causing pro-apoptosis, pro-inflammation and amyloid β-protein cascades (de la Monte, 2014). Imbalance between the production of ROS and antioxidant defenses leads to oxidative stress which not only damages cells but also alters signaling pathways (Hurrle and Hsu, 2017). Oxidative stress has been implicated in AD and several studies have reported that it plays an important role in tau hyperphosphorylation and APP-amyloid β-protein accumulation (Huang et al., 2016).

Tau protein, a major microtubule-associated protein in the brain, functions mainly to maintain the stability of microtubules in neurons and other cells as well as facilitate cell differentiation and polarization (Mouchlis et al., 2020). According to the tau hypothesis, hyperphosphorylated tau pairs with other strands of tau protein and then forms NFT in neuronal cell bodies, which eventually induces microtubule dysregulation (Iqbal et al., 2005), causing impaired communication between neurons and even cell death (Bosco et al., 2011; Kametani and Hasegawa, 2018). As mentioned above, insulin resistance causes production of ROS via the activation of the AGE/RAGE pathway, inducing various stress sensitive signaling pathways, such as NF-κB, JNK/SAPK, p38 MAPK, and Akt pathway in particular (Rains and Jain, 2011). Increased oxidative stress inactivates the Akt pathway, concomitantly to downstream activation of GSK3 and subsequent hyperphosphorylation of tau protein (Bloch-Damti and Bashan, 2005; Hambright et al., 2015; Zhao et al., 2017; Ciotti et al., 2020).

Insulin resistance is also involved in APP-amyloid β-protein accumulation. APP-amyloid β-protein toxic fibrils, in turn, impair insulin signaling by downregulating IRs (Lee et al., 2013). Metal ions, such as zinc and copper bind to amyloid β-protein peptides and catalyze the production of ROS, which causes oxidative damage affecting both amyloid β-protein peptide and surrounding biomolecules, such as proteins and lipids (Cheignon et al., 2018). Both tau hyperphosphorylation and amyloid β-protein accumulation contribute to the positive feedback mechanism that exacerbates insulin/IGF resistance through increased oxidative stress, neurotoxicity and synaptic dysfunction (Lee et al., 2013).

### Decreased Degradation of Amyloid β-Protein Induced by Insulin Resistance via IDE

Insulin is inactivated by IDE, also known as insulin protease (Manolopoulou et al., 2009; Song et al., 2018). IDE is widely distributed in many organs including liver, pancreas, brain and in diverse cellular compartments (Hulse et al., 2009). Accumulating studies have expanded the list of substrates and potential physiological roles of IDE, which includes degradation of multiple bioactive peptides, such as glucagon, IGF-2, and amyloid β-protein (Tang, 2016).

Amyloid β-protein forms various oligomers, leading to fibrils that then aggregate into plaques (Chen et al., 2017), which interrupt normal brain functions. Furthermore, soluble oligomeric forms of amyloid β-protein are the primary toxic species (Haass and Selkoe, 2007; Selkoe and Hardy, 2016) that have been shown to cause synaptic damage and neuronal cell death in both an APP knock-out mouse model and post-mortem human brains from patients with AD (Ding et al., 2019; Rolland et al., 2020). IDE is able to degrade both extracellular and intracellular amyloid β-protein, which protects against formation of these toxic oligomers. In addition, IDE functions as a “dead-end chaperone,” preventing formation of toxic α-synuclein aggregates which can form a stable complex with amyloid β-protein (Sharma et al., 2015). α-synuclein is implicated in the pathophysiology of AD because high levels of α-synuclein are detected in the CSF of patients with MCI and AD (Twohig et al., 2018; Twohig and Nielsen, 2019).

Because insulin and amyloid β-protein are competing substrates for IDE, IDE defects are not only involved in the development of AD but also the basis for a strong association between T2DM and AD. Hyperinsulinemia may downregulate insulin uptake across the BBB and reduce levels of insulin in the brain because of saturation at supraphysiological levels (Reitz and Mayeux, 2014). This may result in decreased levels of IDE (Abdul-Hay et al., 2011; Protzek et al., 2016; Kang et al., 2017), causing decreased degradation of amyloid β-protein and increased deposits of amyloid β-protein (Li et al., 2018). In addition, increased levels of IDE are detected in post-mortem human brains from patients with moderate stage AD (Braak 3–4) whereas significantly reduced level of IDE are found in severe AD (Braak 5–6) (Delikkaya et al., 2019), suggesting that IDE is affected by insulin deficiency and insulin resistance in the early and moderate stages of AD. The development of IDE modulators may be a novel therapeutic approach to both T2DM and AD (Pivovarova et al., 2016).

## Potential Treatments of Insulin Resistance in AD

Potential drug therapies for AD based on the association between insulin resistance and AD are listed in Table 1.

**TABLE 1:** Various potential treatments for Alzheimer’s disease with insulin resistance.

Drug		Classification	Benefits
Anti-diabetic drugs	Metformin	Biguanide	First-line medication for T2DM; anti-inflammation; ↓ Aβ aggregation
	Liraglutide	GLP-1 agonist	^↑^ Insulin secretion; ↓ Aβ accumulation and ↓ tau hyperphosphorylation
	Intranasal insulin	–	Crosses BBB, improves cognitive functions and memory
Anti-inflammatory drugs	Tolfenamic Acid	Fenamate NSAIDs	Anti-inflammation via inhibition of NF-κB pathway; cognition enhancement via↓ Aβ and tau phosphorylation
	Mefenamic Acid	Fenamate NSAIDs	Anti-inflammation via inhibition of NLRP3 inflammasome; improve Aβ-induced learning and memory impairments
	Etanercept	TNF-α inhibitors	Anti-inflammation; ↓ Aβ to ↓ risk of AD
Antioxidant drugs	Vitamin C and E	Antioxidant	↓ Neuronal loss and Aβ; ↓ oxidative stress and tau-induced neurotoxicity
Thiazolidinediones (TZDs)	Rosiglitazone	–	^↑^ Insulin sensitivity; ↓ Aβ levels; improves cognitive functions
	Pioglitazone	–	^↑^ Insulin sensitivity; ↓ Aβ levels via downregulation of APP and BACE1

### Anti-diabetic Drugs

Metformin, a biguanide antihyperglycemic agent which is the first-line medication for T2DM, attenuates inflammation, reduces risk of metabolic syndrome (Li et al., 2015) and may decrease risk of dementia and improve cognitive function. A meta-analysis showed that metformin was beneficial to diabetes patients with dementia or AD (Lin et al., 2018). Interestingly, T2DM patients with long-term use of metformin have been reported to slightly increase the risk of AD (Imfeld et al., 2012) due to metformin-induced vitamin B12 deficiency (Aroda et al., 2016; Campbell et al., 2018). Vitamin B12 deficiency has been reported to increase risk of AD, although the mechanism behind this association is uncertain (Abyad, 2002; Health Quality Ontario., 2013).

Liraglutide, a glucagon-like peptide-1 (GLP-1) receptor agonist, is used to treat T2DM and obesity by increasing insulin release from the pancreas as well as decreasing excessive glucagon release (Femminella et al., 2019). Recent studies have indicated that liraglutide may attenuate cognitive impairment. *In vitro* investigation has shown that liraglutide regulates neuronal insulin signaling and BACE-1 activity to suppress accumulation of amyloid β-protein and hyperphosphorylation of tau protein (Jantrapirom et al., 2020). Also, it prevents loss of brain insulin receptors and synapses and reverses cognitive impairment induced by amyloid β-protein oligomers in mouse hippocampi (Batista et al., 2018).

Intranasal insulin provides a potential pharmacological strategy to treat AD. Although there are different routes of administration for insulin, such as subcutaneous, intramuscular, and oral (Henkin, 2010), intranasal insulin has the advantage of penetrating the BBB and accessing the CNS because of the direct neuroanatomical connections between the olfactory nerves and the brain (de la Monte, 2013) which are beneficial for treating neurodegenerative and psychiatric disease (Hanson and Frey, 2008). More and more clinical studies have shown that intranasal insulin effectively improves cognitive function and memory (Benedict et al., 2008; Hallschmid et al., 2008; Krug et al., 2010), although a newly released study contradicts this finding (Craft et al., 2020). Thus, more direct experimental and clinical evidence are needed to investigate the safety and efficacy of intranasal insulin.

### Anti-inflammatory Drugs

In 2020, 18% of agents in Phase III trials and 15% of agents in Phase II trials targeted inflammation to treat AD (Cummings et al., 2020). This is because a number of epidemiologic studies have reported that anti-inflammatory medication lowers the risk of cognitive impairment and AD. Although the effect of non-steroidal anti-inflammatory drugs (NSAIDs) in AD is under debate (Wang J. et al., 2015; Zhang et al., 2018), fenamate NSAIDS have aroused people’s attention. These compounds selectively inhibit the NLRP3 inflammasome, which is implicated in inflammatory diseases including AD and T2DM, via the inhibition of volume-regulated anion channels (VRACs). The anti-inflammatory effects of two drugs in this class, tolfenamic acid and mefenamic acid, showed benefits in a 3 × TgAD transgenic model of AD (Daniels et al., 2016).

TNF is a key pro-inflammatory cytokine involved in insulin resistance, systemic inflammation and upregulation of amyloid β-protein, which further affects tau hyperphosphorylation (Clark and Vissel, 2015, 2016). Considering the importance of TNF in T2DM and AD pathogenesis, are TNF inhibitors a promising approach to treat AD or AD with T2DM? Although insufficient data are available, TNF inhibitors have been shown to produce cognitive improvements and lower the risk of AD in clinical trials of infliximab and adalimumab (Shi et al., 2011; Zhou et al., 2020). Etanercept, a specific anti-TNF biological in wide clinical use (Clark and Vissel, 2021), has been reported to attenuate neuroinflammation and improve cognitive function in murine models of traumatic brain injury (Chio et al., 2010) and Japanese encephalitis virus (Ye et al., 2014) and in clinical studies (Chen et al., 2010). However, further investigations to evaluate the use and specificity of these agents for dementia needs to be conducted.

### Antioxidant Drugs

Oxidative stress is involved in the pathogenesis of both AD and T2DM. Vitamins C and E, potent antioxidants, are believed to lower the risk of AD and dementia (Lam et al., 2016). This hypothesis is supported by a cohort study which showed a significant protective effect of combined vitamin C and E supplements on cognitive functions in elderly men (Masaki et al., 2000). Another study with 4,740 participants also showed that long-term use of vitamin C and E supplements in combination helped to reduce the incidence of AD (Zandi et al., 2004). In addition, lower plasma levels of vitamin C and E were detected in patients with MCI compared to controls (Rinaldi et al., 2003). However, other studies indicated that vitamins C and E did not reduce the risk of developing AD and vitamin E supplementation had no significant effect on the amyloidotic phenotype if the amyloid plaques were already deposited (Feng and Wang, 2012).

### Thiazolidinediones (TZDs)

The peroxisome proliferator-activated receptor-γ (PPAR- γ), highly expressed in adipose tissue, has a pivotal role in regulating carbohydrate, protein, and lipid metabolism and inflammatory responses (de la Monte, 2017). Thiazolidinediones (TZDs) are synthetic PPAR- γ agonists and potent insulin sensitizers, approved to treat T2DM. TZDs are now considered an attractive treatment of AD because of their potential benefit in cognitive function and memory (Khan et al., 2019). Here, we discuss two prototype TZDs—rosiglitazone and pioglitazone.

Rosiglitazone not only increases insulin sensitivity but also regulates APP processing, leading to reduced plasma amyloid β-protein levels (Pardeshi et al., 2017). Rosiglitazone upregulates IDE levels and downregulates amyloid β-protein levels in a mixed transgenic APPSwe/PS1 mouse model exhibiting both AD and T2DM (Li et al., 2018). Patients with mild to moderate AD in clinical trials were found to significantly improve cognitive function when administrated rosiglitazone (Watson et al., 2005; Risner et al., 2006). However, a phase III trial of rosiglitazone showed no significant effect on cognition (Gold et al., 2010) and rosiglitazone had no effect on the risk of dementia in T2DM patients (Tseng, 2019).

Pioglitazone has been found to increase insulin sensitivity, downregulate levels of hippocampal amyloid β-protein oligomer and decrease pro-cognitive effects in insulin-resistant rats (Yin et al., 2013; Gad et al., 2015). Furthermore, pioglitazone improved cognitive performance in some patients with AD and T2DM (Hanyu et al., 2009; Sato et al., 2011). However, the adverse effects of TZDs, including edema and congestive heart failure, are major limitations for their use in the treatment of dementia and AD (Campbell et al., 2018).

## Discussion

AD is a well-known neurodegenerative disorder, which afflicts millions of people worldwide and places a huge financial burden on society (Jia et al., 2018). For decades, treatments targeting amyloid β-protein based on the amyloid-cascade hypothesis and oligomer-cascade hypothesis have failed (Morris et al., 2014, 2018; Panza et al., 2019). The FDA’s approval of the amyloid β-antibody Aducanumab reflects a promising achievement in AD therapy despite uncertainty about this drug’s clinical benefits and adverse reactions. Apart from amyloid targets, in 2020, according to the FDA registry, there were over 50 agents in clinical trials targeting tau protein, inflammation and metabolism (Cummings et al., 2020). Therefore, novel approaches based on recent insights into this disease are needed.

The role of insulin in AD pathogenesis has only recently gained attention. Insulin resistance may not be the primary cause of AD but it definitely exacerbates AD progression (Clark and Vissel, 2018). In this review, we summarize the mechanisms whereby insulin resistance worsens amyloid β-protein accumulation and tau hyperphosphorylation, including activation of neuroinflammation, activation of oxidative stress and downregulation of IDE. We highlight how insulin resistance and AD form a feed-forward loop in which insulin resistance increases the risk of AD and AD, in turn, exacerbates insulin resistance. Targeting insulin resistance may be a breakthrough strategy to treat AD and may avoid the pitfalls of past treatments targeting amyloid β-protein and tau protein. This review adds to the literature linking insulin resistance and AD by extending insights in this area to update the list of drug candidates that can be repurposed for AD. Further research into the mechanism of the metabolic drivers of AD is needed to identify novel therapeutic approaches for this devastating disease.

## Author Contributions

ZW and JK wrote the first draft of the manuscript. SR conceived the idea for the article and edited the manuscript. All authors contributed to the article and approved the submitted version.

## Conflict of Interest

The authors declare that the research was conducted in the absence of any commercial or financial relationships that could be construed as a potential conflict of interest.

## References

[B1] Abdul-Hay S. O. Kang D. McBride M. Li L. Zhao J. Leissring M. A. (2011). Deletion of insulin-degrading enzyme elicits antipodal, age-dependent effects on glucose and insulin tolerance. *PLoS One* 6:e20818. 10.1371/journal.pone.0020818 21695259 PMC3111440

[B2] Abyad A. (2002). Prevalence of vitamin B12 deficiency among demented patients and cognitive recovery with cobalamin replacement. *J. Nutr. Heal. Aging* 6 254–260. 12486445

[B3] Ahn J.-H. So S.-P. Kim N.-Y. Kim H.-J. Yoon S.-Y. Kim D.-H. (2016). c-Jun N-terminal Kinase (JNK) induces phosphorylation of amyloid precursor protein (APP) at Thr668, in okadaic acid-induced neurodegeneration. *BMB Rep.* 49 376–381. 10.5483/bmbrep.2016.49.7.246 26839154 PMC5032005

[B4] Ardura-Fabregat A. Boddeke E. W. G. M. Boza-Serrano A. Brioschi S. Castro-Gomez S. Ceyzériat K. (2017). Targeting neuroinflammation to treat Alzheimer’s disease. *CNS Drugs* 31 1057–1082. 10.1007/s40263-017-0483-3 29260466 PMC5747579

[B5] Aroda V. R. Edelstein S. L. Goldberg R. B. Knowler W. C. Marcovina S. M. Orchard T. J. (2016). Long-term metformin use and Vitamin B12 deficiency in the diabetes prevention program outcomes study. *J. Clin. Endocrinol. Metab.* 101 1754–1761. 10.1210/jc.2015-3754 26900641 PMC4880159

[B6] Batista A. F. Forny-Germano L. Clarke J. R. Lyra E Silva N. M. Brito-Moreira J. Boehnke S. E. (2018). The diabetes drug liraglutide reverses cognitive impairment in mice and attenuates insulin receptor and synaptic pathology in a non-human primate model of Alzheimer’s disease. *J. Pathol.* 245 85–100. 10.1002/path.5056 29435980 PMC5947670

[B7] Bekris L. M. Yu C. E. Bird T. D. Tsuang D. W. (2010). Review article: genetics of Alzheimer disease. *J. Geriatr. Psychiatry Neurol.* 23 213–227. 10.1177/0891988710383571 21045163 PMC3044597

[B8] Benedict C. Kern W. Schultes B. Born J. Hallschmid M. (2008). Differential sensitivity of men and women to anorexigenic and memory-improving effects of intranasal insulin. *J. Clin. Endocrinol. Metab.* 93 1339–1344. 10.1210/jc.2007-2606 18230654

[B9] Bloch-Damti A. Bashan N. (2005). Proposed mechanisms for the induction of insulin resistance by oxidative stress. *Antioxid. Redox Signal.* 7 1553–1567. 10.1089/ars.2005.7.1553 16356119

[B10] Bosco D. Fava A. Plastino M. Montalcini T. Pujia A. (2011). Possible implications of insulin resistance and glucose metabolism in Alzheimer’s disease pathogenesis. *J. Cell. Mol. Med.* 15 1807–1821. 10.1111/j.1582-4934.2011.01318.x 21435176 PMC3918038

[B11] Boucher J. Kleinridders A. Kahn C. R. (2014). Insulin receptor signaling in normal and insulin-resistant states. *Cold Spring Harb. Perspect. Biol.* 6:a009191. 10.1101/cshperspect.a009191 24384568 PMC3941218

[B12] Brabazon F. Bermudez S. Shaughness M. Khayrullina G. Byrnes K. R. (2018). The effects of insulin on the inflammatory activity of BV2 microglia. *PLoS One* 13:e0201878. 10.1371/journal.pone.0201878 30148836 PMC6110462

[B13] Cai C. Dai X. Zhu Y. Lian M. Xiao F. Dong F. (2016). A specific RAGE-binding peptide biopanning from phage display random peptide library that ameliorates symptoms in amyloid β peptide-mediated neuronal disorder. *Appl. Microbiol. Biotechnol.* 100 825–835. 10.1007/s00253-015-7001-7 26496918

[B14] Campbell J. M. Stephenson M. D. de Courten B. Chapman I. Bellman S. M. Aromataris E. (2018). Metformin use associated with reduced risk of dementia in patients with diabetes: a systematic review and meta-analysis. *J. Alzheimers. Dis.* 65 1225–1236. 10.3233/JAD-180263 30149446 PMC6218120

[B15] Cheignon C. Tomas M. Bonnefont-Rousselot D. Faller P. Hureau C. Collin F. (2018). Oxidative stress and the amyloid beta peptide in Alzheimer’s disease. *Redox Biol.* 14 450–464. 10.1016/j.redox.2017.10.014 29080524 PMC5680523

[B16] Chen G.-F. Xu T.-H. Yan Y. Zhou Y.-R. Jiang Y. Melcher K. (2017). Amyloid beta: structure, biology and structure-based therapeutic development. *Acta Pharmacol. Sin.* 38 1205–1235. 10.1038/aps.2017.28 28713158 PMC5589967

[B17] Chen Y. M. Chen H. H. Lan J. L. Chen D. Y. (2010). Improvement of cognition, a potential benefit of anti-TNF therapy in elderly patients with rheumatoid arthritis. *Joint Bone Spine* 77 366–367. 10.1016/j.jbspin.2010.01.017 20478733

[B18] Cheng X. Shen Y. Li R. (2014). Targeting TNF: a therapeutic strategy for Alzheimer’s disease. *Drug Discov. Today* 19 1822–1827. 10.1016/j.drudis.2014.06.029 24998784

[B19] Chio C. C. Lin J. W. Chang M. W. Wang C. C. Kuo J. R. Yang C. Z. (2010). Therapeutic evaluation of etanercept in a model of traumatic brain injury. *J. Neurochem.* 115 921–929. 10.1111/j.1471-4159.2010.06969.x 20796174

[B20] Ciotti S. Iuliano L. Cefalù S. Comelli M. Mavelli I. Di Giorgio E. (2020). GSK3β is a key regulator of the ROS-dependent necrotic death induced by the quinone DMNQ. *Cell Death Dis.* 11:2. 10.1038/s41419-019-2202-0 31919413 PMC6952365

[B21] Clark I. A. Vissel B. (2015). Amyloid β: one of three danger-associated molecules that are secondary inducers of the proinflammatory cytokines that mediate Alzheimer’s disease. *Br. J. Pharmacol.* 172 3714–3727. 10.1111/bph.13181 25939581 PMC4523330

[B22] Clark I. A. Vissel B. (2016). Excess cerebral TNF causing glutamate excitotoxicity rationalizes treatment of neurodegenerative diseases and neurogenic pain by anti-TNF agents. *J. Neuroinflammation* 13:236. 10.1186/s12974-016-0708-2 27596607 PMC5011997

[B23] Clark I. A. Vissel B. (2018). Therapeutic implications of how TNF links apolipoprotein E, phosphorylated tau, α-synuclein, amyloid-β and insulin resistance in neurodegenerative diseases. *Br. J. Pharmacol.* 175 3859–3875. 10.1111/bph.14471 30097997 PMC6151331

[B24] Clark I. A. Vissel B. (2021). Broader insights into understanding tumor necrosis factor and neurodegenerative disease pathogenesis infer new therapeutic approaches. *J. Alzheimers. Dis.* 79 931–948. 10.3233/JAD-201186 33459706 PMC7990436

[B25] Colombo A. Bastone A. Ploia C. Sclip A. Salmona M. Forloni G. (2009). JNK regulates APP cleavage and degradation in a model of Alzheimer’s disease. *Neurobiol. Dis.* 33 518–525. 10.1016/j.nbd.2008.12.014 19166938

[B26] Craft S. Peskind E. Schwartz M. W. Schellenberg G. D. Raskind M. Porte D. (1998). Cerebrospinal fluid and plasma insulin levels in Alzheimer’s disease: relationship to severity of dementia and apolipoprotein E genotype. *Neurology* 50 164–168. 10.1212/WNL.50.1.164 9443474

[B27] Craft S. Raman R. Chow T. W. Rafii M. S. Sun C. K. Rissman R. A. (2020). Safety, efficacy, and feasibility of intranasal insulin for the treatment of mild cognitive impairment and Alzheimer disease dementia: a randomized clinical trial. *JAMA Neurol.* 77 1099–1109. 10.1001/jamaneurol.2020.1840 32568367 PMC7309571

[B28] Crous-Bou M. Minguillón C. Gramunt N. Molinuevo J. L. (2017). Alzheimer’s disease prevention: from risk factors to early intervention. *Alzheimers Res. Ther.* 9:71. 10.1186/s13195-017-0297-z 28899416 PMC5596480

[B29] Cummings J. Lee G. Ritter A. Sabbagh M. Zhong K. (2020). Alzheimer’s disease drug development pipeline: 2020. *Alzheimers Dement. Transl. Res. Clin. Interv.* 6:e12050. 10.1002/trc2.12050 32695874 PMC7364858

[B30] Daniels M. J. D. Rivers-Auty J. Schilling T. Spencer N. G. Watremez W. Fasolino V. (2016). Fenamate NSAIDs inhibit the NLRP3 inflammasome and protect against Alzheimer’s disease in rodent models. *Nat. Commun.* 7:12504. 10.1038/ncomms12504 27509875 PMC4987536

[B31] de la Monte S. M. (2013). Intranasal insulin therapy for cognitive impairment and neurodegeneration: current state of the art. *Expert Opin. Drug Deliv.* 10 1699–1709. 10.1517/17425247.2013.856877 24215447 PMC4551402

[B32] de la Monte S. M. (2014). Type 3 diabetes is sporadic Alzheimer’s disease: mini-review. *Eur. Neuropsychopharmacol.* 24 1954–1960. 10.1016/j.euroneuro.2014.06.008 25088942 PMC4444430

[B33] de la Monte S. M. (2017). Insulin resistance and neurodegeneration: progress towards the development of new therapeutics for Alzheimer’s Disease. *Drugs* 77 47–65. 10.1007/s40265-016-0674-0 27988872 PMC5575843

[B34] Decourt B. Lahiri D. K. Sabbagh M. N. (2017). Targeting tumor necrosis factor alpha for Alzheimer’s disease. *Curr. Alzheimer Res.* 14 412–425. 10.2174/1567205013666160930110551 27697064 PMC5328927

[B35] Delikkaya B. Moriel N. Tong M. Gallucci G. de la Monte S. M. (2019). Altered expression of insulin-degrading enzyme and regulator of calcineurin in the rat intracerebral streptozotocin model and human apolipoprotein E-ε4-associated Alzheimer’s disease. *Alzheimers Dement. (Amst.)* 11 392–404. 10.1016/j.dadm.2019.03.004 31193223 PMC6522644

[B36] Ding Y. Zhao J. Zhang X. Wang S. Viola K. L. Chow F. E. (2019). Amyloid beta oligomers target to extracellular and intracellular neuronal synaptic proteins in Alzheimer’s Disease. *Front. Neurol.* 10:1140. 10.3389/fneur.2019.01140 31736856 PMC6838211

[B37] Fan Z. Brooks D. J. Okello A. Edison P. (2017). An early and late peak in microglial activation in Alzheimer’s disease trajectory. *Brain* 140 792–803. 10.1093/brain/aww349 28122877 PMC5837520

[B38] Femminella G. D. Frangou E. Love S. B. Busza G. Holmes C. Ritchie C. (2019). Evaluating the effects of the novel GLP-1 analogue liraglutide in Alzheimer’s disease: study protocol for a randomised controlled trial (ELAD study). *Trials* 20:191. 10.1186/s13063-019-3259-x 30944040 PMC6448216

[B39] Feng Y. Wang X. (2012). Antioxidant therapies for Alzheimer’s disease. *Oxid. Med. Cell. Longev.* 2012:472932. 10.1155/2012/472932 22888398 PMC3410354

[B40] Gabbouj S. Ryhänen S. Marttinen M. Wittrahm R. Takalo M. Kemppainen S. (2019). Altered insulin signaling in Alzheimer’s disease brain–special emphasis on PI3K-Akt pathway. *Front. Neurosci.* 13:629. 10.3389/fnins.2019.00629 31275108 PMC6591470

[B41] Gad E. S. Zaitone S. A. Moustafa Y. M. (2015). Pioglitazone and exenatide enhance cognition and downregulate hippocampal beta amyloid oligomer and microglia expression in insulin-resistant rats. *Can. J. Physiol. Pharmacol.* 94 819–828. 10.1139/cjpp-2015-0242 27389824

[B42] Gil-Bea F. J. Solas M. Solomon A. Mugueta C. Winblad B. Kivipelto M. (2010). Insulin levels are decreased in the cerebrospinal fluid of women with prodomal Alzheimer’s disease. *J. Alzheimers Dis.* 22 405–413. 10.3233/JAD-2010-100795 20847404

[B43] Go M. Kou J. Lim J.-E. Yang J. Fukuchi K.-I. (2016). Microglial response to LPS increases in wild-type mice during aging but diminishes in an Alzheimer’s mouse model: Implication of TLR4 signaling in disease progression. *Biochem. Biophys. Res. Commun.* 479 331–337. 10.1016/j.bbrc.2016.09.073 27641666 PMC5048480

[B44] Gold M. Alderton C. Zvartau-Hind M. Egginton S. Saunders A. M. Irizarry M. (2010). Rosiglitazone monotherapy in mild-to-moderate Alzheimer’s disease: results from a randomized, double-blind, placebo-controlled phase III study. *Dement. Geriatr. Cogn. Disord.* 30 131–146. 10.1159/000318845 20733306 PMC3214882

[B45] Guglielmotto M. Aragno M. Tamagno E. Vercellinatto I. Visentin S. Medana C. (2012). AGEs/RAGE complex upregulates BACE1 via NF-κB pathway activation. *Neurobiol. Aging* 33:196.e13-27. 10.1016/j.neurobiolaging.2010.05.026 20638753

[B46] Haass C. Selkoe D. J. (2007). Soluble protein oligomers in neurodegeneration: lessons from the Alzheimer’s amyloid β-peptide. *Nat. Rev. Mol. Cell Biol.* 8 101–112. 10.1038/nrm2101 17245412

[B47] Hallschmid M. Benedict C. Schultes B. Born J. Kern W. (2008). Obese men respond to cognitive but not to catabolic brain insulin signaling. *Int. J. Obes.* 32 275–282. 10.1038/sj.ijo.0803722 17848936

[B48] Hambright H. G. Meng P. Kumar A. P. Ghosh R. (2015). Inhibition of PI3K/AKT/mTOR axis disrupts oxidative stress-mediated survival of melanoma cells. *Oncotarget* 6 7195–7208. 10.18632/oncotarget.3131 25749517 PMC4466678

[B49] Hanson L. R. Frey W. H. II (2008). Intranasal delivery bypasses the blood-brain barrier to target therapeutic agents to the central nervous system and treat neurodegenerative disease. *BMC Neurosci.* 9(Suppl. 3):S5. 10.1186/1471-2202-9-S3-S5 19091002 PMC2604883

[B50] Hanyu H. Sato T. Kiuchi A. Sakurai H. Iwamoto T. (2009). Pioglitazone improved cognition in a pilot study on patients with Alzheimer’s disease and mild cognitive impairment with diabetes mellitus. *J. Am. Geriatr. Soc.* 57 177–179. 10.1111/j.1532-5415.2009.02067.x 19170800

[B51] Havrankova J. Schmechel D. Roth J. Brownstein M. (1978). Identification of insulin in rat brain. *Proc. Natl. Acad. Sci. U.S.A.* 75 5737–5741. 10.1073/pnas.75.11.5737 364489 PMC393044

[B52] Health Quality Ontario. (2013). Vitamin B12 and cognitive function: an evidence-based analysis. *Ont. Health Technol. Assess. Ser.* 13 1–45. PMC3874776 24379897

[B53] Henkin R. I. (2010). Inhaled insulin-Intrapulmonary, intranasal, and other routes of administration: mechanisms of action. *Nutrition* 26 33–39. 10.1016/j.nut.2009.08.001 20005465

[B54] Heron M. (2013). Deaths: leading causes for 2010. *Natl. Vital Stat. Rep.* 62 1–96. 24364902

[B55] Heydemann A. (2016). An overview of murine high fat diet as a model for type 2 Diabetes mellitus. *J. Diabetes Res.* 2016:2902351. 10.1155/2016/2902351 27547764 PMC4983380

[B56] Hsu D. Marshall G. A. (2017). Primary and secondary prevention trials in alzheimer disease: looking back, moving forward. *Curr. Alzheimer Res.* 14 426–440. 10.2174/1567205013666160930112125 27697063 PMC5329133

[B57] Huang W.-J. Zhang X. Chen W.-W. (2016). Role of oxidative stress in Alzheimer’s disease. *Biomed. Rep.* 4 519–522. 10.3892/br.2016.630 27123241 PMC4840676

[B58] Hulse R. E. Ralat L. A. Wei-Jen T. (2009). Structure, function, and regulation of insulin-degrading enzyme. *Vitam. Horm.* 80 635–648. 10.1016/S0083-6729(08)00622-5 19251053 PMC3656499

[B59] Hurrle S. Hsu W. H. (2017). The etiology of oxidative stress in insulin resistance. *Biomed. J.* 40 257–262. 10.1016/j.bj.2017.06.007 29179880 PMC6138814

[B60] Imfeld P. Bodmer M. Jick S. S. Meier C. R. (2012). Metformin, other antidiabetic drugs, and risk of Alzheimer’s disease: a population-based case-control study. *J. Am. Geriatr. Soc.* 60 916–921. 10.1111/j.1532-5415.2012.03916.x 22458300

[B61] Iqbal K. Del C. Alonso A. Chen S. Chohan M. O. El-Akkad E. (2005). Tau pathology in Alzheimer disease and other tauopathies. *Biochim. Biophys. Acta–Mol. Basis Dis.* 1739 198–210. 10.1016/j.bbadis.2004.09.008 15615638

[B62] Jantrapirom S. Nimlamool W. Chattipakorn N. Chattipakorn S. Temviriyanukul P. Inthachat W. (2020). Liraglutide suppresses tau hyperphosphorylation, amyloid beta accumulation through regulating neuronal insulin signaling and BACE-1 activity. *Int. J. Mol. Sci.* 21:1725. 10.3390/ijms21051725 32138327 PMC7084306

[B63] Jha N. K. Jha S. K. Kar R. Nand P. Swati K. Goswami V. K. (2019). Nuclear factor-kappa β as a therapeutic target for Alzheimer’s disease. *J. Neurochem.* 150 113–137. 10.1111/jnc.14687 30802950

[B64] Jia J. Wei C. Chen S. Li F. Tang Y. Qin W. (2018). The cost of Alzheimer’s disease in China and re-estimation of costs worldwide. *Alzheimer’s Dement.* 14 483–491. 10.1016/j.jalz.2017.12.006 29433981

[B65] Johnson L. A. Torres E. R. S. Impey S. Stevens J. F. Raber J. (2017). Apolipoprotein E4 and insulin resistance interact to impair cognition and alter the epigenome and metabolome. *Sci. Rep.* 7:43701. 10.1038/srep43701 28272510 PMC5341123

[B66] Kametani F. Hasegawa M. (2018). Reconsideration of amyloid hypothesis and tau hypothesis in Alzheimer’s disease. *Front. Neurosci.* 12:25. 10.3389/fnins.2018.00025 29440986 PMC5797629

[B67] Kandimalla R. Thirumala V. Reddy P. H. (2017). Is Alzheimer’s disease a type 3 diabetes? a critical appraisal. *Biochim. Biophys. Acta Mol. Basis Dis.* 1863 1078–1089. 10.1016/j.bbadis.2016.08.018 27567931 PMC5344773

[B68] Kang S. Lee Y. H. Lee J. E. (2017). Metabolism-centric overview of the pathogenesis of Alzheimer’s disease. *Yonsei Med. J.* 58 479–488. 10.3349/ymj.2017.58.3.479 28332351 PMC5368131

[B69] Khan M. A. Alam Q. Haque A. Ashafaq M. Khan M. J. Ashraf G. M. (2019). Current progress on peroxisome proliferator-activated receptor gamma agonist as an emerging therapeutic approach for the treatment of Alzheimer’s disease: an update. *Curr. Neuropharmacol.* 17 232–246. 10.2174/1570159X16666180828100002 30152284 PMC6425074

[B70] Kitazawa M. Oddo S. Yamasaki T. R. Green K. N. LaFerla F. M. (2005). Lipopolysaccharide-induced inflammation exacerbates tau pathology by a cyclin-dependent kinase 5-mediated pathway in a transgenic model of Alzheimer’s disease. *J. Neurosci.* 25 8843–8853. 10.1523/JNEUROSCI.2868-05.2005 16192374 PMC6725603

[B71] Kroner Z. (2009). The relationship between Alzheimer’s disease and diabetes: type 3 diabetes? *Altern. Med. Rev.* 14 373–379. 20030463

[B72] Krug R. Benedict C. Born J. Hallschmid M. (2010). Comparable sensitivity of postmenopausal and young women to the effects of intranasal insulin on food intake and working memory. *J. Clin. Endocrinol. Metab.* 95 E468–E472. 10.1210/jc.2010-0744 20719831

[B73] Lam V. Hackett M. Takechi R. (2016). Antioxidants and dementia risk: consideration through a cerebrovascular perspective. *Nutrients* 8:828. 10.3390/nu8120828 27999412 PMC5188481

[B74] Lee D. C. Rizer J. Selenica M.-L. B. Reid P. Kraft C. Johnson A. (2010). LPS- induced inflammation exacerbates phospho-tau pathology in rTg4510 mice. *J. Neuroinflammation* 7:56. 10.1186/1742-2094-7-56 20846376 PMC2949628

[B75] Lee J. W. Lee Y. K. Yuk D. Y. Choi D. Y. Ban S. B. Oh K. W. (2008). Neuro-inflammation induced by lipopolysaccharide causes cognitive impairment through enhancement of beta-amyloid generation. *J. Neuroinflammation* 5:37. 10.1186/1742-2094-5-37 18759972 PMC2556656

[B76] Lee S. Tong M. Hang S. Deochand C. de la Monte S. (2013). CSF and brain indices of insulin resistance, oxidative stress and neuro-inflammation in early versus late Alzheimer’s disease. *J. Alzheimer’s Dis. Park.* 3:128. 10.4172/2161-0460.1000128 25035815 PMC4096626

[B77] Lee S.-H. Zabolotny J. M. Huang H. Lee H. Kim Y.-B. (2016). Insulin in the nervous system and the mind: functions in metabolism, memory, and mood. *Mol. Metab.* 5 589–601. 10.1016/j.molmet.2016.06.011 27656397 PMC5021669

[B78] Leibson C. L. Rocca W. A. Hanson V. A. Cha R. Kokmen E. O’Brien P. C. (1997). Risk of dementia among persons with diabetes mellitus: a population- based cohort study. *Am. J. Epidemiol.* 145 301–308. 10.1093/oxfordjournals.aje.a009106 9054233

[B79] Li H. Wu J. Zhu L. Sha L. Yang S. Wei J. (2018). Insulin degrading enzyme contributes to the pathology in a mixed model of type 2 diabetes and Alzheimer’s disease: possible mechanisms of IDE in T2D and AD. *Biosci. Rep.* 38:BSR20170862. 10.1042/BSR20170862 29222348 PMC6435468

[B80] Li X. Song D. Leng S. X. (2015). Link between type 2 diabetes and Alzheimer’s disease: from epidemiology to mechanism and treatment. *Clin. Interv. Aging* 10 549–560. 10.2147/CIA.S74042 25792818 PMC4360697

[B81] Li X. H. Lv B. L. Xie J. Z. Liu J. Zhou X. W. Wang J. Z. (2012a). AGEs induce Alzheimer-like tau pathology and memory deficit via RAGE-mediated GSK-3 activation. *Neurobiol. Aging* 33 1400–1410. 10.1016/j.neurobiolaging.2011.02.003 21450369

[B82] Li X. H. Xie J. Z. Jiang X. Lv B. L. Cheng X. S. Du L. L. (2012b). Methylglyoxal induces tau hyperphosphorylation via promoting ages formation. *NeuroMolecular Med.* 14 338–348. 10.1007/s12017-012-8191-0 22798221

[B83] Liaoi Y. F. Wang B. J. Cheng H. T. Kuo L. H. Wolfe M. S. (2004). Tumor necrosis factor-α, interleukin-1β, and interferon-γ stimulate γ-secretase-mediated cleavage of amyloid precursor protein through a JNK-dependent MAPK pathway. *J. Biol. Chem.* 279 49523–49532. 10.1074/jbc.M402034200 15347683

[B84] Lin Y. Wang K. Ma C. Wang X. Gong Z. Zhang R. (2018). Evaluation of metformin on cognitive improvement in patients with non-dementia vascular cognitive impairment and abnormal glucose metabolism. *Front. Aging Neurosci.* 10:227. 10.3389/fnagi.2018.00227 30100873 PMC6074058

[B85] Luchsinger J. A. Tang M. X. Stern Y. Shea S. Mayeux R. (2001). Diabetes mellitus and risk of Alzheimer’s disease and dementia with stroke in a multiethnic cohort. *Am. J. Epidemiol.* 154 635–641. 10.1093/aje/154.7.635 11581097

[B86] Manolopoulou M. Guo Q. Malito E. Schilling A. B. Tang W.-J. (2009). Molecular basis of catalytic chamber-assisted unfolding and cleavage of human insulin by human insulin-degrading enzyme. *J. Biol. Chem.* 284 14177–14188. 10.1074/jbc.M900068200 19321446 PMC2682866

[B87] Masaki K. H. Losonczy K. G. Izmirlian G. Foley D. J. Ross G. W. Petrovitch H. (2000). Association of vitamin E and C supplement use with cognitive function and dementia in elderly men. *Neurology* 54 1265–1272. 10.1212/WNL.54.6.1265 10746596

[B88] Matthews K. A. Xu W. Gaglioti A. H. Holt J. B. Croft J. B. Mack D. (2019). Racial and ethnic estimates of Alzheimer’s disease and related dementias in the United States (2015–2060) in adults aged = 65 years. *Alzheimers Dement.* 15 17–24. 10.1016/j.jalz.2018.06.3063 30243772 PMC6333531

[B89] McAlpine F. E. Tansey M. G. (2008). Neuroinflammation and tumor necrosis factor signaling in the pathophysiology of Alzheimer’s disease. *J. Inflamm. Res.* 1 29–39. 10.2147/jir.s4397 22096345 PMC3218716

[B90] McCaulley M. E. Grush K. A. (2017). Seeking a New Paradigm for Alzheimer’s Disease: Considering the Roles of Inflammation, Blood-Brain Barrier Dysfunction, and Prion Disease. *Int. J. Alzheimers. Dis.* 2017:2438901. 10.1155/2017/2438901 29359063 PMC5735673

[B91] Montgomery S. L. Mastrangelo M. A. Habib D. Narrow W. C. Knowlden S. A. Wright T. W. (2011). Ablation of TNF-RI/RII expression in Alzheimer’s disease mice leads to an unexpected enhancement of pathology: implications for chronic pan-TNF-α suppressive therapeutic strategies in the brain. *Am. J. Pathol.* 179 2053–2070. 10.1016/j.ajpath.2011.07.001 21835156 PMC3181376

[B92] Morris G. P. Clark I. A. Vissel B. (2014). Inconsistencies and controversies surrounding the amyloid hypothesis of Alzheimer’s disease. *Acta Neuropathol. Commun.* 2:135. 10.1186/s40478-014-0135-5 25231068 PMC4207354

[B93] Morris G. P. Clark I. A. Vissel B. (2018). Questions concerning the role of amyloid-β in the definition, aetiology and diagnosis of Alzheimer’s disease. *Acta Neuropathol.* 136 663–689. 10.1007/s00401-018-1918-8 30349969 PMC6208728

[B94] Mouchlis V. D. Melagraki G. Zacharia L. C. Afantitis A. (2020). Computer-aided drug design of β-secretase, γ-secretase and anti-tau inhibitors for the discovery of novel alzheimer’s therapeutics. *Int. J. Mol. Sci.* 21:703. 10.3390/ijms21030703 31973122 PMC7038192

[B95] Mullins R. J. Diehl T. C. Chia C. W. Kapogiannis D. (2017). Insulin resistance as a link between amyloid-beta and tau pathologies in Alzheimer’s disease. *Front. Aging Neurosci.* 9:118. 10.3389/fnagi.2017.00118 28515688 PMC5413582

[B96] O’Brien R. J. Wong P. C. (2011). Amyloid precursor protein processing and Alzheimer’s disease. *Annu. Rev. Neurosci.* 34 185–204. 10.1146/annurev-neuro-061010-113613 21456963 PMC3174086

[B97] Panza F. Lozupone M. Seripa D. Imbimbo B. P. (2019). Amyloid-β immunotherapy for Alzheimer disease: is it now a long shot? *Ann. Neurol.* 85 303–315. 10.1002/ana.25410 30635926

[B98] Pardeshi R. Bolshette N. Gadhave K. Ahire A. Ahmed S. Cassano T. (2017). Insulin signaling: an opportunistic target to minify risk of Alzheimer’s disease. *Psychoneuroendocrinology* 83 159–171. 10.1016/j.psyneuen.2017.05.004 28624654

[B99] Peng J. H. Zhang C. E. Wei W. Hong X. P. Pan X. P. Wang J. Z. (2007). Dehydroevodiamine attenuates tau hyperphosphorylation and spatial memory deficit induced by activation of glycogen synthase kinase-3 in rats. *Neuropharmacology* 52 1521–1527. 10.1016/j.neuropharm.2007.02.008 17434540

[B100] Pivovarova O. Höhn A. Grune T. Pfeiffer A. F. H. Rudovich N. (2016). Insulin-degrading enzyme: new therapeutic target for diabetes and Alzheimer’s disease? *Ann. Med.* 48 614–624. 10.1080/07853890.2016.1197416 27320287

[B101] Plum L. Schubert M. Brüning J. C. (2005). The role of insulin receptor signaling in the brain. *Trends Endocrinol. Metab.* 16 59–65. 10.1016/j.tem.2005.01.008 15734146

[B102] Protzek A. O. P. Rezende L. F. Costa-Júnior J. M. Ferreira S. M. Cappelli A. P. G. de Paula F. M. (2016). Hyperinsulinemia caused by dexamethasone treatment is associated with reduced insulin clearance and lower hepatic activity of insulin-degrading enzyme. *J. Steroid Biochem. Mol. Biol.* 155(Pt. A) 1–8. 10.1016/j.jsbmb.2015.09.020 26386462

[B103] Rains J. L. Jain S. K. (2011). Oxidative stress, insulin signaling, and diabetes. *Free Radic. Biol. Med.* 50 567–575. 10.1016/j.freeradbiomed.2010.12.006 21163346 PMC3557825

[B104] Razay G. Wilcock G. K. (1994). Hyperinsulinaemia and Alzheimer’s disease. *Age Ageing* 23 396–399. 10.1093/ageing/23.5.396 7825486

[B105] Reitz C. Mayeux R. (2014). Alzheimer disease: epidemiology, diagnostic criteria, risk factors and biomarkers. *Biochem. Pharmacol.* 88 640–651. 10.1016/j.bcp.2013.12.024 24398425 PMC3992261

[B106] Rinaldi P. Polidori M. C. Metastasio A. Mariani E. Mattioli P. Cherubini A. (2003). Plasma antioxidants are similarly depleted in mild cognitive impairment and in Alzheimer’s disease. *Neurobiol. Aging* 24 915–919. 10.1016/S0197-4580(03)00031-9 12928050

[B107] Risner M. E. Saunders A. M. Altman J. F. B. Ormandy G. C. Craft S. Foley I. M. (2006). Efficacy of rosiglitazone in a genetically defined population with mild-to-moderate Alzheimer’s disease. *Pharmacogenomics J.* 6 246–254. 10.1038/sj.tpj.6500369 16446752

[B108] Rivera E. J. Goldin A. Fulmer N. Tavares R. Wands J. R. De La Monte S. M. (2005). Insulin and insulin-like growth factor expression and function deteriorate with progression of Alzheimer’s disease: link to brain reductions in acetylcholine. *J. Alzheimers Dis.* 8 247–268. 10.3233/JAD-2005-8304 16340083

[B109] Rolland M. Powell R. Jacquier-Sarlin M. Boisseau S. Reynaud-Dulaurier R. Martinez-Hernandez J. (2020). Effect of Aβ oligomers on neuronal APP triggers a vicious cycle leading to the propagation of synaptic plasticity alterations to healthy neurons. *J. Neurosci.* 40 5161–5176. 10.1523/JNEUROSCI.2501-19.2020 32444385 PMC7329309

[B110] Sachdeva A. K. Dharavath R. N. Chopra K. (2019). Time-response studies on development of cognitive deficits in an experimental model of insulin resistance. *Clin. Nutr.* 38 1447–1456. 10.1016/j.clnu.2018.06.966 30037709

[B111] Saini V. (2010). Molecular mechanisms of insulin resistance in type 2 diabetes mellitus. *World J. Diabetes* 1 68–75. 10.4239/wjd.v1.i3.68 21537430 PMC3083885

[B112] Sarlus H. Heneka M. T. (2017). Microglia in Alzheimer’s disease. *J. Clin. Invest.* 127 3240–3249. 10.1172/JCI90606 28862638 PMC5669553

[B113] Sato T. Hanyu H. Hirao K. Kanetaka H. Sakurai H. Iwamoto T. (2011). Efficacy of PPAR-γ agonist pioglitazone in mild Alzheimer disease. *Neurobiol. Aging* 32 1626–1633. 10.1016/j.neurobiolaging.2009.10.009 19923038

[B114] Selkoe D. J. Hardy J. (2016). The amyloid hypothesis of Alzheimer’s disease at 25 years. *EMBO Mol. Med.* 8 595–608. 10.15252/emmm.201606210 27025652 PMC4888851

[B115] Sharma S. K. Chorell E. Steneberg P. Vernersson-Lindahl E. Edlund H. Wittung-Stafshede P. (2015). Insulin-degrading enzyme prevents α-synuclein fibril formation in a nonproteolytical manner. *Sci. Rep.* 5:12531. 10.1038/srep12531 26228656 PMC4521159

[B116] Shi J. Q. Wang B. R. Jiang W. W. Chen J. Zhu Y. W. Zhong L. L. (2011). Cognitive improvement with intrathecal administration of infliximab in a woman with Alzheimer’s disease. *J. Am. Geriatr. Soc.* 59 1142–1144. 10.1111/j.1532-5415.2011.03445.x 21668921

[B117] Song E. S. Rodgers D. W. Hersh L. B. (2018). Insulin-degrading enzyme is not secreted from cultured cells. *Sci. Rep.* 8:2335. 10.1038/s41598-018-20597-6 29402917 PMC5799172

[B118] Soto M. Cai W. Konishi M. Kahn C. R. (2019). Insulin signaling in the hippocampus and amygdala regulates metabolism and neurobehavior. *Proc. Natl. Acad. Sci. U.S.A.* 116 6379–6384. 10.1073/pnas.1817391116 30765523 PMC6442573

[B119] Stanley M. Macauley S. L. Holtzman D. M. (2016). Changes in insulin and insulin signaling in Alzheimer’s disease: cause or consequence? *J. Exp. Med.* 213 1375–1385. 10.1084/jem.20160493 27432942 PMC4986537

[B120] Steen E. Terry B. M. Rivera E. J. Cannon J. L. Neely T. R. Tavares R. (2005). Impaired insulin and insulin-like growth factor expression and signaling mechanisms in Alzheimer’s disease–is this type 3 diabetes? *J. Alzheimers Dis.* 7 63–80. 10.3233/JAD-2005-7107 15750215

[B121] Tang W. J. (2016). Targeting insulin-degrading enzyme to treat type 2 Diabetes mellitus. *Trends Endocrinol. Metab.* 27 24–34. 10.1016/j.tem.2015.11.003 26651592 PMC4698235

[B122] Tokarz V. L. MacDonald P. E. Klip A. (2018). The cell biology of systemic insulin function. *J. Cell Biol.* 217 2273–2289. 10.1083/jcb.201802095 29622564 PMC6028526

[B123] Tseng C.-H. (2019). Rosiglitazone has a neutral effect on the risk of dementia in type 2 diabetes patients. *Aging (Albany. N. Y.).* 11 2724–2734. 10.18632/aging.101944 31085804 PMC6535054

[B124] Twohig D. Nielsen H. M. (2019). α-synuclein in the pathophysiology of Alzheimer’s disease. *Mol. Neurodegener.* 14:23. 10.1186/s13024-019-0320-x 31186026 PMC6558879

[B125] Twohig D. Rodriguez-Vieitez E. Sando S. B. Berge G. Lauridsen C. Møller I. (2018). The relevance of cerebrospinal fluid α-synuclein levels to sporadic and familial Alzheimer’s disease. *Acta Neuropathol. Commun.* 6:130. 10.1186/s40478-018-0624-z 30477568 PMC6260771

[B126] Wang J. Tan L. Wang H. F. Tan C. C. Meng X. F. Wang C. (2015). Anti-inflammatory drugs and risk of Alzheimer’s Disease: an updated systematic review and meta-analysis. *J. Alzheimers Dis.* 44 385–396. 10.3233/JAD-141506 25227314

[B127] Wang W.-Y. Tan M.-S. Yu J.-T. Tan L. (2015). Role of pro-inflammatory cytokines released from microglia in Alzheimer’s disease. *Ann. Transl. Med.* 3:136. 10.3978/j.issn.2305-5839.2015.03.49 26207229 PMC4486922

[B128] Watson G. S. Baker L. D. Cholerton B. A. Rhoads K. W. Merriam G. R. Schellenberg G. D. (2009). Effects of insulin and octreotide on memory and growth hormone in Alzheimer’s disease. *J. Alzheimers Dis.* 18 595–602. 10.3233/JAD-2009-1165 19625744 PMC2842464

[B129] Watson G. S. Cholerton B. A. Reger M. A. Baker L. D. Plymate S. R. Asthana S. (2005). Preserved cognition in patients with early Alzheimer disease and amnestic mild cognitive impairment during treatment with rosiglitazone: a preliminary study. *Am. J. Geriatr. Psychiatry* 13 950–958. 10.1176/appi.ajgp.13.11.950 16286438

[B130] Watson G. S. Craft S. (2003). The role of insulin resistance in the pathogenesis of Alzheimer’s disease: Implications for treatment. *CNS Drugs* 17 27–45. 10.2165/00023210-200317010-00003 12467491

[B131] Wilson R. S. Segawa E. Boyle P. A. Anagnos S. E. Hizel L. P. Bennett D. A. (2012). The natural history of cognitive decline in Alzheimer’s disease. *Psychol. Aging* 27 1008–1017. 10.1037/a0029857 22946521 PMC3534850

[B132] Xie L. Helmerhorst E. Taddei K. Plewright B. Van Bronswijk W. Martins R. (2002). Alzheimer’s beta-amyloid peptides compete for insulin binding to the insulin receptor. *J. Neurosci.* 22:RC221. 10.1523/jneurosci.22-10-j0001.2002 12006603 PMC6757630

[B133] Xu W. L. Von Strauss E. Qiu C. X. Winblad B. Fratiglioni L. (2009). Uncontrolled diabetes increases the risk of Alzheimer’s disease: a population-based cohort study. *Diabetologia* 52 1031–1039. 10.1007/s00125-009-1323-x 19280172

[B134] Ye J. Jiang R. Cui M. Zhu B. Sun L. Wang Y. (2014). Etanercept reduces neuroinflammation and lethality in mouse model of Japanese encephalitis. *J. Infect. Dis.* 210 875–889. 10.1093/infdis/jiu179 24652493

[B135] Yin Q.-Q. Pei J.-J. Xu S. Luo D.-Z. Dong S.-Q. Sun M.-H. (2013). Pioglitazone improves cognitive function via increasing insulin sensitivity and strengthening antioxidant defense system in fructose-drinking insulin resistance rats. *PLoS One* 8:e59313. 10.1371/journal.pone.0059313 23527159 PMC3603906

[B136] Zandi P. P. Anthony J. C. Khachaturian A. S. Stone S. V. Gustafson D. Tschanz J. A. T. (2004). Reduced risk of Alzheimer disease in users of antioxidant vitamin supplements: the cache county study. *Arch. Neurol.* 61 82–88. 10.1001/archneur.61.1.82 14732624

[B137] Zhang C. Wang Y. Wang D. Zhang J. Zhang F. (2018). NSAID exposure and risk of Alzheimer’s disease: an updated meta-analysis from cohort studies. *Front. Aging Neurosci.* 10:83. 10.3389/fnagi.2018.00083 29643804 PMC5882872

[B138] Zhang T. Chen D. Lee T. H. (2019). Phosphorylation signaling in APP processing in Alzheimer’s disease. *Int. J. Mol. Sci.* 21:209. 10.3390/ijms21010209 31892243 PMC6981488

[B139] Zhao W. Q. Townsend M. (2009). Insulin resistance and amyloidogenesis as common molecular foundation for type 2 diabetes and Alzheimer’s disease. *Biochim. Biophys. Acta Mol. Basis Dis.* 1792 482–496. 10.1016/j.bbadis.2008.10.014 19026743

[B140] Zhao Y. Hu X. Liu Y. Dong S. Wen Z. He W. (2017). ROS signaling under metabolic stress: cross-talk between AMPK and AKT pathway. *Mol. Cancer* 16:79. 10.1186/s12943-017-0648-1 28407774 PMC5390360

[B141] Zhou M. Xu R. Kaelber D. C. Gurney M. E. (2020). Tumor Necrosis Factor (TNF) blocking agents are associated with lower risk for Alzheimer’s disease in patients with rheumatoid arthritis and psoriasis. *PLoS One* 15:e0229819. 10.1371/journal.pone.0229819 32203525 PMC7089534

